# Education-Based Gaps in eHealth: A Weighted Logistic Regression Approach

**DOI:** 10.2196/jmir.5188

**Published:** 2016-10-12

**Authors:** Laura Amo

**Affiliations:** ^1^ State University of New York at Buffalo Buffalo, NY United States

**Keywords:** information storage and retrieval, eHealth, models, statistical

## Abstract

**Background:**

Persons with a college degree are more likely to engage in eHealth behaviors than persons without a college degree, compounding the health disadvantages of undereducated groups in the United States. However, the extent to which quality of recent eHealth experience reduces the education-based eHealth gap is unexplored.

**Objective:**

The goal of this study was to examine how eHealth information search experience moderates the relationship between college education and eHealth behaviors.

**Methods:**

Based on a nationally representative sample of adults who reported using the Internet to conduct the most recent health information search (n=1458), I evaluated eHealth search experience in relation to the likelihood of engaging in different eHealth behaviors. I examined whether Internet health information search experience reduces the eHealth behavior gaps among college-educated and noncollege-educated adults. Weighted logistic regression models were used to estimate the probability of different eHealth behaviors.

**Results:**

College education was significantly positively related to the likelihood of 4 eHealth behaviors. In general, eHealth search experience was negatively associated with health care behaviors, health information-seeking behaviors, and user-generated or content sharing behaviors after accounting for other covariates. Whereas Internet health information search experience has narrowed the education gap in terms of likelihood of using email or Internet to communicate with a doctor or health care provider and likelihood of using a website to manage diet, weight, or health, it has widened the education gap in the instances of searching for health information for oneself, searching for health information for someone else, and downloading health information on a mobile device.

**Conclusion:**

The relationship between college education and eHealth behaviors is moderated by Internet health information search experience in different ways depending on the type of eHealth behavior. After controlling for college education, it was found that persons who experienced more fruitful Internet health information searches are generally less likely to engage in eHealth behaviors.

## Introduction

People often turn to the Internet to obtain health-related information [1-4]. As of 2012, 72% of adult Internet users in the United States reported looking online for health-related information [5]. The term *eHealth* emerged in the early 2000s [6,7] with eHealth behaviors defined as online-mediated health self-management behaviors. There is extant research suggesting that eHealth behaviors can help people take better care of themselves and can lead to optimal health outcomes [8-10]. However, while more and more Americans are engaging in different eHealth behaviors, there is evidence of a digital divide across socioeconomic lines. Specifically, college-educated individuals have a higher likelihood of engaging in eHealth behaviors relative to individuals without a college education [11,12]. Thus, the benefits associated with eHealth behaviors may be less accessible to persons without a college degree.

In order for eHealth behaviors to translate into positive health outcomes, a person must have the ability and motivation to (1) find information, (2) understand it, and (3) follow through with the appropriate behaviors. Information foraging [13] is a theory that can help explain how information is acquired; eHealth literacy [14] can be used to explain understanding and comprehension; and psychobehavioral models can be used to explain how information is processed, internalized, and translated into behavior.

Information foraging theory describes information retrieval in terms of cost and benefits [13] and has been used to understand health information-seeking behaviors [15]. Information foraging is based on information value, information patches, information scents, and information diet [16]. According to information foraging theory, foraging persists if information that is retrieved is useful and relevant [13], and an information search is maximized when multiple information sources are utilized, which is particularly pertinent in online environments where a multitude of information sources are readily available.

Information foraging for health information online is interconnected to eHealth literacy. Health literacy is defined as the ability to access and use information about health and medicine [17] to make choices about health care, prevention, and promotion [18,19,20]. More specifically, eHealth literacy is defined as “the ability to seek, find, understand, and appraise health information from electronic sources and apply the knowledge gained to addressing or solving a health problem” [14,21]. Health literacy is related to health outcomes; individuals who are better able to understand and utilize health information tend to experience better health outcomes [22-27] and tend to have higher rates of insurance coverage [2]. However, while health literacy is strongly associated with health outcomes, a large number of Americans are at a disadvantage—as of 2001, it was estimated that about 30 million Americans have below basic health literacy [2].

Nevertheless, in order to develop literacy of any kind, information needs to be gathered first. Information foraging may be conceptualized as a prerequisite to developing literacy, including eHealth literacy, and thus cannot be overlooked when examining eHealth behaviors. However, information foraging has not received attention in the eHealth literature. In this paper, I focus on aspects of the information foraging process in predicting different eHealth behaviors. I examine how recent eHealth search experiences are associated with eHealth behaviors and explore whether the quality of recent eHealth search experiences reduces education-based gaps in eHealth behaviors.

Q1: Do recent eHealth search experiences relate to eHealth behaviors?

Q2: Does eHealth search experience moderate the relationship between college education and eHealth behaviors?

Among most of the existing research studies on eHealth, there are several limitations. First, there tends to be a lack of statistical rigor; with the exception of a few recent studies [11,28], research on eHealth behaviors tends to focus on prevalence (ie, percentage) as opposed to association with eHealth literacy. Causal models are optimal [29,30], and research that explores significant associations with eHealth behaviors and the relationships between health literacy and eHealth behaviors is also important [11]. Another limitation of existing eHealth studies is that researchers tend to assume homogeneity among all Internet users in regard to how the Internet is used for health information; in certain research studies [31,32], Internet users are compared with nonInternet users with the assumption that both groups are uniform. However, there is evidence of a digital divide that extends beyond Internet access [11,33]. The digital divide does not just pertain to access, but also for the purpose and utility of Internet use. Internet users may use the Internet in very different ways to manage health and search for health-related information. In this study, I examine the education-based digital divide by examining how college-educated Internet users differ from noncollege-educated Internet users in terms of eHealth experiences and behaviors.

## Methods

### Data

The data used in this study are from the Health Information National Trends Survey (HINTS) 4, Cycle 1 data collection in 2014 [34]. The HINTS uses a 2-stage stratified random sampling method. See HINTS manual for more information on stratification. In keeping with Kontos et al’s methods, the sample is composed of Internet users. To directly address the construct of eHealth literacy, a filter was also applied such that only persons who had used the Internet for the most recent search of health-related information (n=1458, after list-wise deletion).

### Weighting

**Figure 1:**
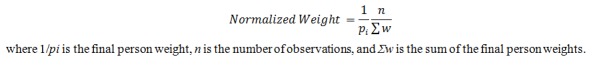
Equation for normalized weight.

The final person weights were calculated in 4 steps: calculating household-level base weight, adjusting for household-level nonresponse, calculating person-level base weights, and calibrating person-level weights to population counts (or control totals). In order to address the nonindependence of observations and design effect, the final person weights were normalized. The final person weights were multiplied by the total number of observations in the analytic sample over the sum of the final person weights (See Figure 1).

### Variables

**Health:** Self-reported rating of health based on a five-point scale where 1=*Poor* and 5=*Excellent*.

**Age (years):** The 4 age groups were *18-34* (reference), *35-49*, *50-64*, *65-74,* and *75 and older.* Each of the age groups was transformed into a dummy variable.

**Salary:** The 5 groups were below US $20000, US $20000-$34999, US $35000-$49999, US $50000-$749999, and US $75000 and over (reference).

**Male:** A dummy code where 1=*male* and 0=*female*.

**Employed:** A dummy code where 1=*employed* and 0=*unemployed*.

**History of Cancer in Family:** A dummy code was created where 1=*yes* and 0=*no*.

**History of personal cancer in lifetime:** A dummy code where 1=*yes* and 0=*no*.

**Insurance coverage:** A dummy code where 1=*yes* and 0=*no*.

**USA birthplace:** A dummy code where 1=*yes* and 0=*no*.

**College degree:** A dummy code indicating whether or not a respondent had a college degree or higher (0=*no*, 1=*yes*).

**Some college:** A dummy code indicating whether or not a respondent had attended but not completed college (0=*no*, 1=*yes*).

**High school or below:** A dummy code indicating whether or not a respondent had a high school degree or below (0=*no*, 1=*yes*).

**Hispanic:** A dummy code where 1=*yes* and 0=*no*.

**Non-Hispanic black:** A dummy code where 1=*yes* and 0=*no*.

**Other race:** A dummy code where 1=*yes* and 0=*no*.

**Non-Hispanic white:** A dummy code where 1=*yes* and 0=*no*. This variable was the reference category for race.

**Married:** A dummy code where 1=*yes* and 0=*no*. This served as a reference category for the single/divorced/widowed variable.

**Single/Divorced/Widowed:** A dummy code where 1=*yes* and 0=*no*.

**Number of children in the household:** The number of children aged 18 years old and younger who were living in the household at the time of survey administration.

**Most recent check-up:** Responses were captured as ordinal data where higher values indicated less recent visits. Specifically, 1=*Within the past year*, 2=*Within the past 2 years*, 3=*Within past 5 years*, 4=*5 or more years ago*, 5=*Don’t know*, and 6=*Never*.

**Frequency of doctor visits within 12 months:** Responses were captured as ordinal data where 0=*None*, 1=*1 time*, 2=*2 times*, 3=*3 times*, 4=*4 times*, 5=*5 – 9 times*, and 6=*10 or more times*. This excluded emergency room (ER) visits.

**Home ownership:** A dummy code where 1=*yes* and 0=*no*.

**Past experiences with eHealth:** Participants had all used the Internet to perform the most recent search of health-related information. In relation to this search, participants were asked to rate the following statements on a five-point scale, where 1=*Strongly Agree* and 5=*Strongly Disagree*: “It took a lot of effort to get the information you needed,” “You felt frustrated during your search for the information,” “You were concerned about the quality of the information,” and “The information you found was hard to understand.” Estimates of internal reliability using Cronbach alpha=.862. Original scoring was retained such that higher scores reflected less effort, less frustration, less confusion, and less difficulty in understanding health-related information as retrieved from the Internet.

**eHealth care behaviors:** Indicated whether or not an individual had (1) bought medicine or vitamins online, (2) looked for a health care provider online, (3) tracked personal health information online, or (4) used email or the Internet to communicate with health care provider. Each of the 4 behaviors was coded such that 0=*no* and 1=*yes*.

**eHealth information behaviors:** Indicated whether or not an individual had (1) searched for health information for themselves, (2) searched for health information for someone else, (3) used a website to manage health or weight, or (4) downloaded health information to a mobile device. Each of the 4 behaviors was coded such that 0=*no* and 1=*yes*.

**Social eHealth behaviors:** Indicated whether or not an individual had (1) used a social networking site to read and share about medical issues, (2) wrote in an online diary or blog that was health-related, and (3) participated in an online support group. Each of the 3 behaviors was coded such that 0=*no* and 1=*yes*.

### Analysis

The analysis consisted of 12 logistic regression models predicting each of the eHealth behaviors characterized by Kontos et al [11]. Logistic regression is a form of regression used when the outcome variable is dichotomous; in this analysis, each of the outcomes is binary where 0=“No” and 1=“Yes.” Assumptions of normality are violated in this type of regression and the outcomes are therefore transformed using the logarithmic transformation [35,36]. Interactions between college education and health literacy were grand-mean centered to reduce multicollinearity [36]. This procedure consisted of finding the mean of both college education and health literacy, subtracting each value from the mean, then multiplying the 2 centered terms to produce a grand-mean centered interaction between college education and health literacy [36]. In addition to reporting the beta values, I also report the odds ratios for each term and the pseudo *R*^2^ for each model. In logistic regression, the two pseudo- *R*^2^ indices are Cox and Snell’s *R*^2^ and Nagelkerke’s *R*^2^ [37].

## Results

### eHealth Care Behaviors

Descriptive statistics for the sample are provided in Table 1, and results from the logistic regressions are provided in Multimedia Appendices 1–3. Internet users were engaged in eHealth care behaviors at a moderate rate: 325 of 1585 Internet users (about 20%) bought medicine or vitamins online; 223 of 1585 (about 14%) looked for health care providers online; 1093 of 1584 (about 69%) tracked personal health information; and 767 of 1585 (about 48%) used email or the Internet to communicate with a doctor or health care provider.

Consistent with prior research, college education was significantly positively related to some of the eHealth care behaviors. Compared with persons who had completed high school or less, persons who had at least a 4-year college degree had significantly higher likelihood of looking for health care providers (beta=.783, *P*=.001) and using email or the Internet to communicate with the doctor (beta=.403, *P*=.006). College education did not have a significant association with likelihood of buying medicine or vitamins online (beta=.181, *P*=.37) or tracking personal health information (beta=.138, *P*=.40).

The relationship between eHealth information search experience and health care behaviors was mostly negative (see Multimedia Appendix 1). Specifically, eHealth information search experience was significantly negatively related to likelihood of buying medicine or vitamins online (beta=-.196, *P*=.04) and tracking personal health information (beta=-.98, *P*=.02). In other words, persons who had more positive and less frustrating past experiences with eHealth information hunting were significantly less likely to use the Internet to buy medicine or vitamins online and to track personal health information online. While eHealth information search experience was negatively related to the likelihood of looking online for health care providers (beta=-.20, *P*=.08), the relationship was not statistically significant with alpha=.05; the quality of previous eHealth information search experiences was not associated with the likelihood of using email or Internet to communicate with a doctor (beta=.011, *P*=.89). In terms of change in odds ratios [35,37], a one standard deviation increase in quality of eHealth information search experience was associated with a .822 decrease in the odds of buying medicine or vitamins online, a .816 decrease in the odds of looking for health care providers, and a .82 decrease in the odds of tracking personal health information.

The quality of eHealth information search experience did not significantly alter the college gap in terms of likelihood of buying medicine or vitamins online (beta=.136, *P*=.46), looking for health care providers (beta=-0.095, *P*=.66), and tracking personal health information (beta=0.290, *P*=.07). However, the quality of eHealth information search experience did significantly reduce the education gap in terms of likelihood to use email or the Internet to communicate with the doctor or health care provider (beta=-.416, *P*=.006). As shown in Multimedia Appendix 1, the full models accounted for between 9-14% of the variance in the likelihood of buying medicine or vitamins online, between 8-15% of the variance in the likelihood of looking for health care providers, between 5-7% of the variance in the likelihood of tracking personal health information online, and between 9-12% of the variance in the likelihood of using email or the Internet to communicate with the doctor.

### eHealth Information Behaviors

Internet users also used the Internet to engage in eHealth information behaviors. Of 1818 Internet users, 1632 (nearly 90%) indicated using the Internet to search for health information for himself or herself; 189 of 1568 Internet users (about 12%) used the Internet to search for health information for someone else; 715 of 1580 (about 45%) used the Internet to access websites to assist with managing diet, weight, or health; and 269 of 1586 (about 15%) downloaded health-related information to a mobile device.

College education was significantly positively related to one health information behavior. Compared with persons who had completed high school or less, persons who had at least a 4-year college degree had significantly higher likelihood of looking for health information for another person (beta=1.125, *P*≤.001). College education was not, however, significantly related to the likelihood of searching for health information for self (beta=.336, *P*=.23), using a website to help with diet, health, or weight (beta=.356, *P*=.006), or to the likelihood of downloading health information to a mobile device (beta=-.143, *P*=.51).

The relationship between eHealth information search experience and health care behaviors was mostly negative. Specifically, more positive eHealth information search experiences were negatively related to likelihood of searching for health information for self (beta=-.288, *P*=.07), looking for health information for another person (beta=-.441, *P*≤.001), and downloading health information to a mobile device (beta=-.033 *P*=.75). The quality of previous eHealth information search experience was positively related to the likelihood of using a website to help with diet, health, or weight (beta=.249, *P*=.1), but the relationship was not statistically significant.

The quality of eHealth information search experience significantly reduced the college gap in terms of using a website to help with diet, weight, or health (beta=-.401, *P*=.009). In other words, persons with a college degree were more likely to use a website to help with diet, weight, or health. If previous eHealth information search experiences had been more positive and less frustrating, the gap between college-educated and noncollege-educated Internet users was significantly reduced.

Interestingly, as opposed to reducing the education-based gap, the quality of eHealth information search experience exacerbated the gap in terms of likelihood of searching for health information for self (beta=.102, *P*=.02), searching for health information for another person (beta=.761, *P*=.001), and downloading health information to a mobile device (beta=.421, *P*=.045). As shown in Multimedia Appendix 2, the full models accounted for (1) between 6-14% of the variance in the likelihood of using the Internet to search for health information for self, (2) between 6-11% of the variance in the likelihood of using the Internet to search for health information for another person, (3) between 9-13% of the variance in the likelihood of using a website to help with diet, health, or weight, and (4) between 8-13% of the variance in the likelihood of downloading health information to a mobile device.

**Table 1:** Descriptive statistics on Internet users (n=2056).

Variables		n	Min	Max	Mean	SD
Health		1819	1	5	3.603	0.907
**Age (years)**						
	18-34 (reference)	1827	0	1	0.356	0.479
	35-49	1827	0	1	0.315	0.465
	50-64	1827	0	1	0.251	0.434
	65-74	1827	0	1	0.053	0.223
	≥75	1827	0	1	0.017	0.129
**Salary (US $)**						
	<20000	1827	0	1	0.164	0.371
	20000 – 34999	1827	0	1	0.131	0.337
	35000 – 49999	1827	0	1	0.104	0.306
	50000 – 74999	1827	0	1	0.179	0.383
	75000 + (reference)	1827	0	1	0.352	0.478
Male		1805	0	1	0.453	0.498
Employed		1827	0	1	0.631	0.483
History of cancer in family		1827	0	1	0.648	0.478
History of cancer in self		1827	0	1	0.057	0.232
Insurance		1827	0	1	0.806	0.395
Born in United States		1827	0	1	0.886	0.318
**Education**						
	High school or below	1827	0	1	0.213	0.410
	Some college	1827	0	1	0.328	0.470
	College or more	1827	0	1	0.447	0.497
**Race or ethnicity**						
	Hispanic	1827	0	1	0.113	0.317
	Non-Hispanic black	1827	0	1	0.088	0.283
	Other race	1827	0	1	0.708	0.455
	Non-Hispanic white (reference)	1827	0	1	0.066	0.248
**Marital status**						
	Married or living as married	1827	0	1	0.566	0.496
	Single divorced, widowed, or separated	1827	0	1	0.419	0.494
Number of children		1777	0	9	0.643	1.036
Most recent check-up		1811	1	6	1.932	1.333
Frequency doctor visit in 12 months		1819	0	6	2.410	1.880
eHealth search experience		1812	−2.36	1.36	−0.034	0.960
Home-owner		1807	0	1	0.610	0.488
Health information for self		1818	0	1	0.898	0.303
Bought medicine or vitamins online		1585	0	1	0.205	0.404
Online support group		1592	0	1	0.055	0.228
Email or Internet to communicate with doctor		1585	0	1	0.484	0.500
Website to help with diet, weight, or physical health		1580	0	1	0.453	0.498
Looked for health care provider		1586	0	1	0.141	0.348
Download health related information to mobile device		1586	0	1	0.170	0.375
Social networking site to read and share about medical		1591	0	1	0.039	0.194
Wrote an health-related online diary or blog		1585	0	1	0.206	0.404
Kept track of personal information		1584	0	1	0.690	0.463
Health information for someone else		1568	0	1	0.120	0.325
Valid N (list-wise)		1458				

### Social eHealth Care Behaviors

According to the results, Internet users demonstrated less use of Internet for social eHealth care behaviors, relative to the other eHealth behaviors. Only 62 of 1591 Internet users (about 4%) indicated using the Internet to participate in a social networking group for health-related reasons; 326 of 1585 (about 21%) indicated writing in an online blog that was health-related; and 88of 1592 (about 6%) participated in an online support group for health-related purposes.

College education was significantly positively related to one of the social health behaviors. Compared with persons who had completed high school or less, persons who had at least a 4-year college degree had significantly higher likelihood of writing in an online diary or participate in a health-related blog (beta=.783, *P*≤.001); people with at least a college degree were twice as likely to engage in this social health care behavior. There was no education gap in terms of the likelihood of using a social networking site to read and share medical-related information (beta=.171, *P*=.71) or participating in an online support group (beta=-.118, *P*=.75).

The relationship between the quality of eHealth information search experience and social health behaviors was all negative, but not statistically significant. Specifically, the quality of eHealth information search experience was negatively related to the likelihood of using a social networking site to read and share medical-related information (beta=-.187, *P*=.36) and participating in an online support group (beta=-.047, *P*=.64), and with the likelihood of writing in an online diary or participating in a health-related blog (beta=-.247, *P*=.14).

The quality of eHealth information search experience did not significantly alter the college gap in terms of likelihood of using a social networking site to read and share medical-related information (beta=-.47, *P*=.25), writing in an online diary or participating in a health-related blog (beta=-.079, *P*=.68), and participating in an online support group (beta=-.404, *P*=.23). As shown in Multimedia Appendix 3, the full models accounted for between 6-22% of the variance in likelihood of using a social networking site to read and share medical information, between 10-17% of the variance in the likelihood of writing in an online diary or participating in a health-related blog, and between 6-19% of the variance in the likelihood of participating in an online support group.

## Discussion

### Principal Findings

Health self-management is a major objective for new health care models such as the Chronic Care model [38]. Ideally, technology will improve decision making surrounding health care and medicine and allow traditionally vulnerable populations to be better informed. However, findings from this study confirm that there are persistent gaps in eHealth behaviors across educational lines. Among people who used the Internet as primary means for searching health-related information, those with at least a 4-year college degree were significantly more likely to engage in at least four of the eHealth behaviors explored in this study (ie, looking for a health care provider, using email or the Internet to communicate with a doctor, searching for health information for someone else, and writing in an online diary or blog for health-related reasons).

Beyond these persistent educational gaps in eHealth behaviors, findings from this study suggest that at least in some instances, these educational gaps are exacerbated when considering how persons feel and react to recent eHealth search experiences. This may mean that college education is not necessarily directly related to eHealth behaviors yet obtaining a college education may provide the ancillary benefit of making persons “better” foragers of eHealth information. In short, there appears to be a complex relationship between college education, eHealth experiences, and eHealth behaviors where additional statistical mediators and moderators are important to probe in future research. Indeed, the quality of recent eHealth experiences was significantly negatively associated with the likelihood of engaging in at least nine of the twelve eHealth behaviors investigated herein. This finding is, at first, quite troubling. However, it makes sense that if a person experiences a high-quality information search and obtains adequate information during the information foraging process, then perhaps certain eHealth behaviors are therefore not necessary.

In 2 instances, quality of recent eHealth information search experience reduced the education-based gap; eHealth search experience reduced the gap between college-educated and noncollege-educated persons in terms of the likelihood of using email or the Internet to communicate with a doctor or physician and use of website to manage diet, weight, or health. The negative moderating effect of eHealth search experience on the education-based gap in using email or the Internet to communicate with the doctor or health care provider may be related to the ways in which health care is being managed on the provider’s end. Currently, there is a trend for doctors and health care providers to use technology for record keeping, appointment making, and patient requests. Another potential explanation is that patients, regardless of college education, find communication via technology to be more convenient and cost saving (ie, not having to go visit the doctor in person).

The other moderating negative effect of eHealth literacy was with regard to use of website to manage diet, weight, or health; persons without a college degree who have higher eHealth literacy are more likely to use a website to manage diet, weight, or health. Overall, this eHealth behavior was fairly prevalent (about 45% of the sample engaged in this behavior) among persons without a college degree who engaged in this behavior, regardless of eHealth literacy.

### Limitations

There were a number of limitations to this study. First, the data are cross-sectional and longitudinal analysis could not be performed. Design of the study is such that casual inference cannot be made. Second, the data are secondary, meaning that omitted variable bias is also an issue. There is no way to control for information that was not included in the primary data collection, such as quality of primary health provider (eg, board certification, quality of education, year MD received), and these types of characteristics are very likely to make a difference in the relationships that were studied.

Despite these limitations, the findings from this study open the door to other questions that need to be explored in the eHealth landscape. There are other sociodemographic variables that need to be addressed in relation to eHealth behaviors, for example, while education-based gaps in eHealth behaviors may not be reduced by quality of recent eHealth search experience as measured herein, racial-based gaps should also be investigated. If persons from a certain social or economic background experience eHealth information searches differently than other groups, this is an important issue to consider. Perhaps most importantly, there is also a need to evaluate temporal models that explore how eHealth search experience and eHealth literacy are associated with different types of eHealth behaviors and how these eHealth behaviors subsequently impact health outcomes; if eHealth behaviors fail to positively relate to health outcomes after controlling for other covariates in the model, eHealth discrepancies between socioeconomic and racial groups may not be problematic. Assuming that eHealth behaviors indeed make a difference and can be influenced by variables amenable to intervention such as eHealth search experience and literacy, future studies should also consider the role of specific types of websites (eg, design, interface, services) that are most commonly used for eHealth behaviors.

**Conclusion**

eHealth is a promising new frontier in health access and care. However, as findings from this study show, there are socioeconomic gaps in eHealth behaviors that may not be easily addressed. Moreover, the quality of eHealth information search experiences may serve to reduce the likelihood of eHealth behaviors. Existing research into eHealth tends to assume that eHealth behaviors are a “good outcome,” and that persons who are not engaging in eHealth behaviors are at a disadvantage. However, this assumption may not be correct. Persons who are not engaging in eHealth behaviors may not have to because of (1) better health, (2) access to other health alternatives or resources that have not been identified in the research, or (3) because previous eHealth information searches were successful and questions were answered. Moving forward, researchers and policy-makers will be better served by framing eHealth behaviors as component to a much larger, extremely complex web of behavior, cognition, social influence, and future health outcomes as opposed to an absolute positive outcome directly related to optimal health for all.

## Appendix

Multimedia Appendix 1 eHealth information search experience and college education: health care behaviors.

Multimedia Appendix 2 eHealth information search experience and college education: health information behaviors.

Multimedia Appendix 3 eHealth information search experience and college education: social health behaviors.

## References

[1] Hsu W, Chiang C, Yang S (2014). J Med Internet Res.

[2] Baker DW, Gazmararian JA, Williams MV, Scott T, Parker RM, Green D, Ren J, Peel J (2002). Functional health literacy and the risk of hospital admission among Medicare managed care enrollees. Am J Public Health.

[3] Licciardone JC, Smith-Barbaro P, Coleridge ST (2001). Use of the Internet as a resources for consumer health information: Results of the Second Osteopathic Survey of Health Care in America (OSTEOSURV-II). J Med Internet Res.

[4] Houston TK, Allison JJ (2002). Users of internet health information: Differences by health status. J Med Internet Res . DOI 10.2196/jmir.4.2.e.

[5] Pew Research Center (2015). pewinternet.

[6] Eng T (2001). The ehealth landscape: a terrain map of emerging information and communication technologies in health and health care.

[7] Eysenbach G (2001). What is e-health?. J Med Internet Res.

[8] Bianco A, Zucco R, Nobile CG, Pileggi C, Pavia M (2013). Parents seeking health-related information on the Internet: cross-sectional study. J Med Internet Res.

[9] Agency for Healthcare Research Quality (2014). ahrq.

[10] Lambert SD, Loiselle CG (2007). Health information seeking behavior. Qual Health Res.

[11] Kontos E, Blake KD, Chou WY, Prestin A (2014). Predictors of eHealth Usage: Insights on the digital divide from the Health Information National Trends Survey 2012. J Med Internet Res.

[12] Diaz JA, Griffith RA, Ng JJ, Reinert SE, Friedmann PD, Moulton AW (2002). Patients' use of the internet for medical information. J Gen Intern Med.

[13] Pirolli P, Card SK (1999). Information foraging. Psychological Review.

[14] Norman CD, Skinner HA (2006). eHealth Literacy: Essential Skills for Consumer Health in a Networked World. J Med Internet Res.

[15] Xiao N, Sharman R, Rao H, Upadhyaya S (2014). Factors influencing online health information search: An empirical analysis of a national cancer-related survey. Decision Support Systems.

[16] Trepess D (2016). interaction-design.

[17] McCray AT (2005). Promoting health literacy. J Am Med Inform Assoc.

[18] World Health Organization (2013). World Health Organization.

[19] Berkman ND, Davis TC, McCormack L (2010). Health literacy: What is it?. Journal of Health Communication: International Perspectives.

[20] Ratzan SC, Parker RM, Selden CR, Zorn M, Ratzan SC, Parker RM (2000). Introduction. National Library of Medicine Current Bibliographies in Medicine: Health Literacy.

[21] Norman CD, Skinner HA (2006). eHEALS: The eHealth Literacy Scale. J Med Internet Res.

[22] Scott Tracy L, Gazmararian Julie A, Williams Mark V, Baker David W (2002). Health literacy and preventive health care use among Medicare enrollees in a managed care organization. Med Care.

[23] Williams MV, Baker DW, Parker RM, Nurss JR (1998). Relationship of functional health literacy to patients' knowledge of their chronic disease. A study of patients with hypertension and diabetes. Arch Intern Med.

[24] Schillinger D, Grumbach K, Piette J, Wang F, Osmond D, Daher C, Palacios J, Sullivan GD, Bindman AB (2002). Association of health literacy with diabetes outcomes. JAMA.

[25] Schillinger D, Piette J, Grumbach K, Wang F, Wilson C, Daher C, Leong-Grotz K, Castro C, Bindman AB (2003). Closing the loop: physician communication with diabetic patients who have low health literacy. Arch Intern Med.

[26] Baker DW, Parker RM, Williams MV, Clark WS, Nurss J (1997). The relationship of patient reading ability to self-reported health and use of health services. Am J Public Health.

[27] Baker DW, Parker RM, Williams MV, Clark WS (1998). Health literacy and the risk of hospital admission. J Gen Intern Med.

[28] Mitsutake Seigo, Shibata Ai, Ishii Kaori, Oka Koichiro (2016). Associations of eHealth Literacy With Health Behavior Among Adult Internet Users. J Med Internet Res.

[29] Nielsen-Bohlman L, Panzer AM, Kindig DA (2004). https://www.nap.edu/read/10883/chapter/1.

[30] Ad Hoc Committee on Health Literacy for the Council on Scientific Affairs American Medical Association (1999). Report of the Council on Scientific Affairs. Journal of American Medical Association.

[31] Fox S (2006). Pew Internet.

[32] Zillien N, Hargittai E (2009). Digital Distinction: Status-Specific Types of Internet Usage. Social Science Quarterly.

[33] Sarkar U, Karter AJ, Liu JY, Adler NE, Nguyen R, López A, Schillinger D (2011). Social disparities in internet patient portal use in diabetes: evidence that the digital divide extends beyond access. J Am Med Inform Assoc.

[34] Nelson DE, Kreps GL, Hesse BW, Croyle RT, Willis G, Arora NK, Rimer BK, Viswanath KV, Weinstein N, Alden S (2004). The Health Information National Trends Survey (HINTS): development, design, and dissemination. J Health Commun.

[35] Berry W, Feldman S (1985). Multiple regression in practice. University Paper Series on Quantitative Approaches in Social Sciences.

[36] Aiken L, West SG (1991). MultipleRegression: Testing and Interpreting Interactions.

[37] Field A (2001). Discovering Statistics Using SPSS. Second edition.

[38] Wagner EH (1998). Chronic disease management: what will it take to improve care for chronic illness?. Eff Clin Pract.

