# Supplementary material for: Education-Based Gaps in eHealth: A Weighted Logistic Regression Approach
Source: J Med Internet Res. 2016 Oct 12;18(10):e267. doi: 10.2196/jmir.5188 (PMC5081480; doi:10.2196/jmir.5188)
Supplement: Multimedia Appendix 1 [file jmir_v18i10e267_app1.pdf]

Table 2A. eHealth Information Search Experience and College Education: Health Care Behaviors

	Bought medicine or vitamins online			Looked for health care provider		
	B(SE)	Exp (beta)	*P*	*B*(SE)	Exp (beta)	*P*
constant	1.906(0.59)	6.729	.001	-0.879(0.69)	.415	.203
Health	-0.209(0.09)	.812	.015	-0.135(0.10)	.873	.173
age35-49	-0.322(0.19)	.725	.094	-0.563(0.22)	.57	.011
age50-64	-0.199(0.21)	.820	.348	-0.384(0.24)	.681	.114
age65-74	0.529(0.31)	1.697	.093	-0.916(0.48)	.400	.059
Age 75 and above	-1.152(0.69)	.316	.093	-1.017(0.74)	.362	.171
Less than US $20000	-1.502(0.29)	.223	<.001	0.813(0.28)	2.254	.003
US $20000 – US $34999	-0.799(0.25)	.450	.001	0.406(0.29)	1.502	.166
US $35000-US $49999	-0.551(0.25)	.577	.030	-0.107(0.34)	.899	.755
US $50000-US $74999	0.048(0.18)	1.049	.791	0.464(0.23)	1.591	.043
Male	-0.095(0.14)	.909	.501	0.156(0.17)	1.168	.359
Employed	0.003(0.16)	1.003	.987	0.092(0.19)	1.096	.622
Family cancer	-0.162(0.15)	.850	.264	0.206(0.18)	1.228	.251
Personal cancer	0.226(0.28)	1.253	.416	-0.361(0.41)	.697	.375
Health Coverage	-0.269(0.22)	.764	.224	-0.280(0.24)	.756	.241
Born in USA	-1.116(0.24)	.328	<.001	-0.440(0.25)	.644	.082
College or more	0.181(0.20)	1.199	.373	0.783(0.24)	2.188	.001
Some college	0.389(0.21)	1.475	.060	-0.021(0.26)	.979	.935
Hispanic	0.313(0.25)	1.367	.203	0.519(0.29)	1.681	.069
Black (non-Hispanic)	0.599(0.24)	1.820	.013	0.538(0.28)	1.713	.054
Other race	-0.695(0.34)	.499	.038	1.41(0.29)	4.096	.000
Single	0.286(0.18)	1.330	.104	-0.184(0.21)	.832	.389
Number of children	-0.165(0.08)	.848	.044	-0.221(0.10)	.802	.032
Most recent check-up	-0.269(0.07)	.764	<.001	-0.159(0.07)	.853	.028
Frequency doctor	-0.062(0.04)	.940	.141	0.108(0.05)	1.114	.028
Own home	0.285(0.18)	1.330	.108	0.110(0.19)	1.117	.571
eHealth Experience	-0.196(0.09)	.822	.038	-0.203(0.12)	.816	.079
College X eHealth Experience	0.136(0.18)	1.146	.459	-0.095(0.22)	.909	.662
Cox & Snell R^2	0.091			0.085		
Nagelkerke R^2	0.142			0.153		

Table 2B: eHealth Information Search Experience and College Education: Health Care Behaviors (cont'd)

	Tracked personal health information			Used email or Internet to communicate with doctor		
	B(SE)	Exp(beta)	*P*	*B*(SE)	Exp(beta)	*P*
constant	1.286(0.51)	3.618	.012	2.034(0.49)	7.642	<.001
Health	0.165(0.07)	1.18	.022	-0.133(0.07)	.875	.048
age35-49	0.388(0.17)	1.474	.019	-0.350(0.15)	.705	.020
age50-64	0.179(0.18)	1.197	.327	-0.582(0.17)	.559	.001
age65-74	-0.134(0.29)	0.875	.643	-1.106(0.29)	0.331	<.001
Age 75 and above	-0.230(0.45)	.794	.610	-2.125(0.53)	.119	<.001
Less than US $20000	0.249(0.21)	1.283	.243	-0.619(0.21)	.539	.003
US $20000 – US $34999	0.591(0.22)	1.806	.007	-0.351(0.20)	.704	.079
US $35000-US $49999	0.138(0.21)	1.147	.515	-0.489(0.20)	.613	.015
US $50000-US $74999	0.090(0.17)	1.094	.59	0.290(0.16)	1.336	.069
Male	-0.237(0.12)	.789	.051	-0.131(0.11)	.877	.250
Employed	-0.140(0.14)	.869	.314	-0.309(0.13)	.734	.017
Family cancer	-0.06(0.13)	.942	.636	-0.126(0.12)	.881	.288
Personal cancer	-0.169(0.25)	.844	.495	0.209(0.24)	1.232	.386
Health Coverage	-0.382(0.19)	.682	.040	-0.36(0.17)	.698	.035
Born in USA	-0.246(0.23)	.782	.295	-0.686(0.22)	.503	.002
College or more	0.138(0.17)	1.148	.404	0.430(0.16)	1.537	.006
Some college	0.121(0.17)	1.128	.472	-0.144(0.16)	.866	.367
Hispanic	-0.123(0.22)	.884	.573	0.102(0.21)	1.107	.625
Black (non-Hispanic)	-0.099(0.22)	.906	.654	-0.019(0.21)	.981	.929
Other race	0.474(0.29)	1.606	.106	-0.302(0.26)	.739	.242
Single	-0.532(0.15)	.587	.000	0.480(0.14)	1.616	.001
Number of children	0.136(0.07)	1.145	.047	0.025(0.06)	1.025	.675
Most recent check-up	-0.034(0.05)	.966	.499	-0.099(0.05)	.906	.038
Frequency doctor	0.118(0.04)	1.125	.001	0.003(0.03)	1.003	.932
Own home	-0.267(0.15)	.766	.072	-0.075(0.14)	.927	.580
eHealth Experience	-0.198(0.08)	.820	.016	0.011(0.08)	1.011	.886
College X eHealth Experience	0.290(0.16)	1.336	.073	-0.416(0.15)	.660	.006
Cox & Snell R^2	0.054			0.092		
Nagelkerke R^2	0.076			0.123		
