# Supplementary material for: Education-Based Gaps in eHealth: A Weighted Logistic Regression Approach
Source: J Med Internet Res. 2016 Oct 12;18(10):e267. doi: 10.2196/jmir.5188 (PMC5081480; doi:10.2196/jmir.5188)
Supplement: Multimedia Appendix 2 [file jmir_v18i10e267_app2.pdf]

Table 3A. eHealth Information Search Experience and College Education: Health Information Behaviors

	Health information for self			Health information for another person		
	B(SE)	Exp (beta)	*P*	*B*(SE)	Exp (beta)	*P*
Constant	4.316(0.75)	74.92	<.001	-1.622(0.77)	.197	.035
health	-0.132(0.07)	.842	.092	-0.167(0.10)	.846	.105
Age 35-49	1.676(0.32)	1.026	.916	0.147(0.23)	1.158	.532
Age 50-64	0.986(0.30)	.409	.001	0.440(0.25)	1.553	.083
Age 65-74	0.542(0.29)	.469	.083	0.307(0.43)	1.360	.470
Age 75 and above	0.297(0.32)	.227	.023	0.578(0.61)	1.782	.347
Less than US $20000	0.152(0.23)	1.753	.093	0.484(0.29)	1.623	.099
US $20000 –$34999	0.043(0.21)	.915	.751	0.157(0.32)	1.170	.619
US $35000-$49999	-0.092(0.20)	1.211	.522	-0.125(0.33)	.882	.704
US $50000-$74999	-0.108(0.17)	1.258	.349	-0.253(0.26)	.776	.327
Male	0.032(0.12)	1.096	.599	-0.664(0.18)	.515	<.001
Employed	0.005(0.14)	.847	.406	-0.074(0.19)	.929	.697
Family cancer	-0.247(0.13)	.865	.423	-0.343(0.18)	.710	.052
Personal cancer	-0.049(0.22)	.858	.662	-0.414(0.38)	.661	.276
Health coverage	-0.425(0.20)	.694	.169	0.170(0.27)	1.185	.531
Born in USA	-0.105(0.22)	.462	.026	0.477(0.34)	1.611	.158
College or more	0.336(0.17)	1.322	.227	1.125(0.27)	3.080	<.001
Some college	-0.051(0.16)	.956	.843	0.258(0.28)	1.294	.360
Hispanic	-0.287(0.20)	.437	.002	-0.042(0.33)	.959	.899
Black (non-Hispanic)	0.045(0.24)	.933	.850	-0.279(0.33)	.756	.403
Other race	-0.346(0.27)	.448	.023	0.029(0.39)	1.029	.940
Single	0.179(0.15)	1.629	.027	0.225(0.22)	1.252	.298
Number of children	0.067(0.07)	1.051	.600	0.122(0.09)	1.130	.164
Most recent check-up	-0.118(0.05)	.806	.001	0.051(0.07)	1.052	.484
Frequency of doctor	0.207(0.04)	1.360	<.001	0.106(0.05)	1.112	.032
Own home	0.249(0.15)	1.257	.274	-0.052(0.21)	.949	.807
eHealth Experience	-0.288(0.08)	.807	.067	-0.441(0.12)	.643	<.001
College X eHealth Experience	0.102(0.16)	1.731	.020	0.761(0.23)	2.141	.001
Cox & Snell R^2	0.068			0.058		
Nagelkerke R^2	0.139			0.111		

Table 3B. eHealth Information Search Experience and College Education: Health Information Behaviors (cont'd)

	Used a website to help with diet, weight, or health			Downloaded health information to mobile		
	B(SE)	Exp (beta)	*P*	*B*(SE)	Exp (beta)	*P*
Constant	1.280(0.49)	3.596	.008	-1.562(0.68)	.21	.022
health	-0.401(0.15)	.670	.009	-0.015(0.09)	.985	.868
Age 35-49	-0.445(0.17)	.641	.010	0.261(0.20)	1.299	.182
Age 50-64	-1.203(0.31)	.300	<.001	-0.281(0.24)	.755	.233
Age 65-74	-0.448(0.45)	.639	.324	-1.114(0.47)	.328	.018
Age 75 and above	0.143(0.20)	1.154	.478	-0.381(0.69)	.683	.580
Less than US $20000	-0.523(0.21)	.593	.012	0.435(0.27)	1.545	.106
US $20000 – US $34999	0.446(0.20)	1.562	.026	1.093(0.25)	2.982	<.001
US $35000-US $49999	0.059(0.16)	1.061	.709	0.456(0.26)	1.578	.079
US $50000-US $74999	-0.251(0.11)	.778	.029	0.181(0.22)	1.199	.412
Male	0.232(0.13)	1.261	.073	-0.461(0.16)	.631	.003
Employed	-0.278(0.12)	.757	.020	-0.090(0.17)	.914	.597
Family cancer	0.110(0.25)	1.116	.655	0.026(0.16)	1.026	.875
Personal cancer	0.179(0.17)	1.196	.298	-0.010(0.34)	.99	.977
Health coverage	-0.265(0.21)	.767	.216	0.082(0.22)	1.085	.709
Born in USA	0.419(0.16)	1.521	.008	0.905(0.34)	2.472	.008
College or more	0.240(0.16)	1.271	.139	-0.143(0.22)	.867	.514
Some college	-0.455(0.21)	.634	.033	0.482(0.21)	1.619	.020
Hispanic	0.582(0.21)	1.789	.006	0.055(0.27)	1.056	.842
Black (non-Hispanic)	0.706(0.26)	2.026	.007	-0.376(0.28)	.687	.176
Other race	-0.488(0.15)	.614	.001	0.356(0.36)	1.428	.324
Single	0.115(0.06)	1.122	.057	-0.451(0.19)	.637	.018
Number of children	-0.033(0.05)	.967	.485	-0.052(0.08)	.949	.517
Most recent check-up	0.078(0.03)	1.082	.020	-0.313(0.07)	.731	<.001
Frequency of doctor	-0.252(0.14)	.777	.069	0.046(0.04)	1.047	.295
Own home	-0.305(0.08)	.737	<.001	-0.558(0.18)	.572	.002
eHealth Experience	0.249(0.15)	1.283	.099	-0.033(0.10)	.967	.748
College X eHealth Experience	-0.401(0.15)	.670	.009	0.421(0.21)	1.523	.045
Cox & Snell R^2	0.094			0.079		
Nagelkerke R^2	0.125			0.133		
