# Supplementary material for: Education-Based Gaps in eHealth: A Weighted Logistic Regression Approach
Source: J Med Internet Res. 2016 Oct 12;18(10):e267. doi: 10.2196/jmir.5188 (PMC5081480; doi:10.2196/jmir.5188)
Supplement: Multimedia Appendix 3 [file jmir_v18i10e267_app3.pdf]

Table 4A. eHealth Information Search Experience and College Education: Social Health Behaviors

	Social networking site to read and share about medical			Wrote an online diary or blog health-related		
	B(SE)	Exp (beta)	*P*	*B*(SE)	Exp (beta)	*P*
Constant	-3.518(1.43)	.030	.014	-2.667(0.66)	.069	<.001
Health	0.090(0.18)	1.094	.620	0.018(0.09)	1.018	.833
Age 35-49	-0.507(0.38)	.602	.186	-0.415(0.19)	.660	.031
Age 50-64	-0.899(0.46)	.407	.053	-0.487(0.21)	.614	.023
Age 65-74	-1.603(0.91)	.201	.078	-0.187(0.33)	.829	.569
Age 75 and above	-18.591(7548.85)	.000	.998	-0.113(0.49)	.893	.816
Less than US $20000	0.130(0.57)	1.139	.819	0.468(0.26)	1.598	.072
US $20000 – US $34999	1.188(0.46)	3.281	.010	0.085(0.27)	1.088	.751
US $35000-US $49999	-0.829(0.78)	.436	.286	-0.155(0.28)	.856	.575
US $50000-US $74999	0.362(0.41)	1.436	.382	0.668(0.18)	1.951	<.001
Male	-2.224(0.50)	.108	<.001	-0.339(0.15)	.712	.020
Employed	-0.229(0.33)	.795	.483	0.241(0.17)	1.272	.145
Family cancer	0.322(0.34)	1.380	.346	0.032(0.15)	1.032	.832
Personal cancer	0.916(0.50)	2.499	.070	0.183(0.28)	1.201	.511
Health coverage	0.203(0.50)	1.225	.682	1.370(0.31)	3.936	<.001
Born in United States	0.359(0.63)	1.431	.570	-0.270(0.26)	.763	.295
College or more	0.171(0.45)	1.186	.708	0.783(0.21)	2.187	<.001
Some college	0.544(0.44)	1.724	.213	0.351(0.23)	1.421	.119
Hispanic	-0.544(0.65)	.581	.405	-0.031(0.27)	.970	.909
Black (non-Hispanic)	-0.743(0.71)	.476	.292	-0.052(0.27)	.949	.845
Other race	0.632(0.61)	1.882	.299	0.442(0.30)	1.555	.139
Single	-0.997(0.40)	.369	.012	0.005(0.18)	1.005	.977
Number of children	-0.050(0.16)	.951	.748	-0.038(0.08)	.963	.627
Most recent check-up	0.097(0.12)	1.102	.416	-0.243(0.07)	.784	<.001
Frequency of visiting doctor	0.244(0.08)	1.276	.003	0.157(0.04)	1.170	<.001
Own home	-0.267(0.36)	.765	.463	-0.195(0.18)	.823	.276
eHealth Experience	-0.187(0.20)	.829	.360	-0.047(0.10)	.954	.638
College X eHealth Experience	-0.470(0.40)	.625	.245	-0.079(0.19)	.924	.675
Cox & Snell R^2	0.063			0.107		
Nagelkerke R^2	0.225			0.168		

Table 4B. eHealth Information Search Experience and College Education: Social Health Behaviors (cont'd)

	Participated in an online support group		
	B(SE)	Exp(beta)	*P*
Constant	-0.817(1.02)	.442	.424
Health	-0.227(0.15)	.797	.133
Age 35-49	-0.429(0.31)	.651	.173
Age 50-64	-0.640(0.38)	.527	.090
Age 65-74	-0.479(0.65)	.619	.460
Age 75 and above	-18.671(7809.94)	.000	.998
Less than US $20000	-0.662(0.46)	.516	.148
US $20000-US $35000	0.157(0.40)	1.171	.696
US $35000-US $50000	-0.279(0.50)	.756	.577
US $50000-US $75000	1.152(0.32)	3.166	<.001
Male	-1.098(0.29)	.334	<.001
Employed	0.228(0.27)	1.256	.400
Family cancer	0.547(0.29)	1.729	.063
Personal cancer	-0.182(0.59)	.834	.756
Health coverage	-0.851(0.36)	.427	.018
Born in United States	-1.194(0.40)	.303	.003
College or more	-0.118(0.37)	.889	.748
Some college	0.841(0.35)	2.319	.015
Hispanic	0.088(0.41)	1.092	.831
Black (non-Hispanic)	-1.525(0.66)	.218	.021
Other race	0.047(0.53)	1.048	.928
Single	0.458(0.30)	1.58	.130
Number of children	-0.047(0.14)	.954	.745
Most recent check-up	0.004(0.10)	1.004	.969
Frequency of visiting doctor	0.202(0.07)	1.224	.005
Own home	0.341(0.30)	1.406	.249
eHealth Experience	-0.247(0.17)	.781	.135
College X eHealth Experience	-0.404(0.34)	.667	.233
Cox & Snell R^2	0.067		
Nagelkerke R^2	0.189		
